# It’s time to talk fathers: The impact of paternal depression on parenting style and child development during the COVID-19 pandemic

**DOI:** 10.3389/fpsyg.2022.1044664

**Published:** 2022-11-21

**Authors:** Joshua Paul Roberts, Rose-Marie Satherley, Jane Iles

**Affiliations:** School of Psychology, University of Surrey, Guildford, United Kingdom

**Keywords:** child development, COVID-19, paternal depression, paternal parenting, parenting, lockdown, parenting style

## Abstract

This study aimed to understand the relationship between paternal depression, parenting behavior and child developmental outcomes during the SARS-CoV-2 (COVID) pandemic. In addition, the paternal experience of the pandemic, such as the impact of lockdowns, was explored. Fathers of children aged 6–11 years old (*n* = 87) were recruited for an online cross-sectional survey. Data was collected through questionnaires and open-ended comments. Regression analysis indicated a higher level of self-reported depressive symptomology in fathers more severely impacted by the pandemic across financial, familial and health domains. Further, COVID-19 impact, but not paternal depression, was linked to fewer authoritative parenting behaviors, characterized as lower warmth and responsiveness. Paternal pandemic impact and depression symptoms were independently predictive of child cognitive scores, and both were associated with emotional and behavioral outcomes. A content analysis of open-ended responses from fathers noted that concerns for their children, work and mental health were most prevalent during the pandemic. However, several responders also reported no change or positive facets of lockdowns related to the pandemic. These finds are discussed in the context of a possible behavioural mechanism of action accounting for the effect of these factors on child development. Clinical implications include targeted interventions for at risk groups as well as psychoeducation for fathers that acknowledge difference in paternal coping and support seeking.

## Introduction

The advent of empirical research exploring child development brought with it a focus on the maternal-child dyad. However, the familial landscape has changed. Paternal time given to caregiving is thought to have almost tripled over the last 50 years, from approximately 5–13%, taken as an average per week (Davison et al., 2017). The conceptualization of fatherhood has also shifted, from disciplinarian breadwinner to nurturing caregiver (Sarkadi et al., 2008; Yogman and Garfield, 2016). In developmental terms, the presence of the father during a child’s upbringing has been associated with positive child outcomes including academic performance, psychological adjustment, behavior, and emotional regulation (Roggman et al., 2004; Cabrera et al., 2007; McMunn et al., 2017). Despite this, fathers remain generally under-represented in family research (Davison et al., 2017; Cabrera et al., 2018).

To build a better understanding of child development, the paternal influence must also be explored. A growing body of research indicates paternal play is more likely to involve physicality, competitiveness, and unpredictability, whereas mothers have been found to orientate toward object-use, explicit learning, and verbal interaction (Fletcher et al., 2011; John et al., 2013; Robinson et al., 2021). Each play style is important, but disparately linked to developmental skills (Cabrera et al., 2014; Jeynes, 2016). For instance, paternal parenting behaviors have been linked to child cognitive abilities, including executive function (Papaleontiou-Louca and Omari, 2020). The children of fathers demonstrating sensitivity, warmth, and stimulation, particularly during paternal play, have been associated with better cognitive and linguistical outcomes aged 2–5 years old (Mills-Koonce et al., 2015; Rolle et al., 2019). Such associations warrant further exploration in terms of the explicit paternal behaviors which may underpin this relationship, as well as what may occur when external factors impact the parenting role.

The negative effect of maternal depressive symptomology on child development has been well documented (e.g., Liu et al., 2017; Fredrikson et al., 2019). A recent meta-analysis reported depression in mothers was associated with lower cognition scores in children aged 5 years or younger, after adjusting for confounding variables, such as household income and maternal education (Liu et al., 2017). Child behavioral and socio-emotional difficulties have also been linked with maternal depressive symptoms, across the childhood lifespan (Villodas et al., 2015; Waerden et al., 2015; Charrois et al., 2020). Further, the relationship between depressive symptomology in mothers and child developmental outcomes is seemingly mediated by a change in parenting style, specifically less warmth, stimulation, sensitivity, and responsiveness (Liu et al., 2017; Baker and Kuhn, 2018).

However, research addressing paternal depression on child outcomes remains insufficient despite it effecting up to almost 10% of fathers 1-year postpartum (Glasser and Lerner-Geva, 2018). The existing literature does suggest paternal depression may impact parenting behaviors, such as increased anger and irritability, as well as reduced levels of warmth, stimulation, sensitivity, and responsiveness (Sethna et al., 2015; O’Brien et al., 2017). Perinatal paternal depression has been associated with difficulties in vocabulary learning, higher rates of emotional and behavioral difficulties in pre-school age children, and psychopathology and emotional difficulties across the lifespan (Sweeney and MacBeth, 2016; Gentile and Fusco, 2017). However, there is a paucity of studies exploring the potential impact on offspring cognitive outcomes (Rolle et al., 2019). Further, little is known regarding how to specifically support depressed fathers. Given the recent global pandemic, the gaps in research pertaining to paternal depression are of particular significance.

The public health crisis resulting from the spread of SARS-CoV-2 (COVID-19) has potentially changed the paternal parenting landscape once again. Global lockdowns over a 2-year period resulted in a potentially uniquely high level of paternal influence over child development (Cito et al., 2020). As previously outlined, increased father involvement has the potential to enhance infant outcomes, however, concurrent with the increase in paternal presence was additional financial, workplace and childcare stress (Patrick et al., 2020; Cheng et al., 2021). Early research has indicated that global lockdowns had a detrimental impact on parental mental health and wellbeing, yet the specific changes caused by the pandemic in the lives of parents remains unknown (Cheng et al., 2021; Martiny et al., 2021; Schmidt et al., 2021).

This study aimed to better understand the relationship between the impact of COVID-19 and paternal depression, as well as associations with parenting behavior and child developmental outcomes (child cognition, behavior, and emotional difficulties). The previous literature pertaining to paternal depression and child development has largely focused on younger infants. Due to the emphasis on child cognition fathers of children aged 6–11 years old were recruited, in-line with the concrete-operational stage of child learning (Feldman, 2005; Waber et al., 2007; Blair, 2016). A further aim was to explore key qualitative pandemic-related change for fathers. These aims were established in the hope of providing information which could be used to improve support for fathers and families following an unprecedented public health crisis.

It was hypothesized that greater COVID-19 impact would be associated with higher scores of paternal depressive symptoms. In addition, paternal depressive symptoms would correlate with less positive parenting behaviors, such as warmth and autonomy granting. Finally, depressive symptomology in fathers would predict lower scores on measures of child cognition, and behavioral and emotional outcomes.

## Materials and methods

This study employed a cross-sectional survey to understand the relationships between COVID-19, paternal mental health, parenting behaviors and child developmental outcomes. The survey was conducted online between April 2021 and January 2022, during the global COVID-19 pandemic.

### Participants

Fathers were eligible to take part if they identified as the father or stepfather of a child between 6 and 11 years old and if they resided with said child at the time of participation and had done predominantly throughout their upbringing. Fathers were excluded from participation if they had been absent from the family home for more than six consecutive months and if their child had a diagnosed learning disability or neurodevelopmental disorder.

Participants were recruited *via* online advertising, including social media and parenting forums. Schools across the UK also disseminated the survey to fathers by email and posters. Interested fathers were directed to an online link, which provided further information. Consenting participants then completed the online survey through Qualtrics Software, Provo, UT (2021), a secure online data collection platform. Completion of the survey took approximately 10–20 min. After survey completion, a standardized debrief was provided to each participant, explaining the purpose of the study with links to relevant advice and resources. Fathers were offered the opportunity to win a £50 Amazon voucher as recompense for their participation.

### Measures

Demographic information was gathered on father and child’s age and gender (Table 1). If there was more than one child aged 6–11 years old within the family, fathers were asked to answer all questions based on their oldest child. Paternal ethnic background was requested, as well as social economic data, highest qualification attained and annual household income. GCSE and NVQ qualifications represent secondary education. A bachelor’s degree is attained at university, and this alongside post-graduation and doctoral degrees are considered higher education.

**TABLE 1:** Participant demographic information.

Factor	Fathers (%)
**Ethnicity**	
Asian (Indian, Pakistani, Bangladeshi, Chinese)	6 (7)
Black (African, British, Caribbean)	3 (4)
White (British, Irish)	70 (82)
Other/mixed background	6 (7)
**Highest education**	
GCSE	5 (6)
NVQ	7 (8)
A-Level	8 (9)
Bachelor’s degree	30 (35)
Post-graduate	29 (34)
Doctorate	7 (8)
**Household income**	
£0 – £39,999	15 (17)
£40,000 – £79,999	35 (40)
£80,000 – £100,000+	37 (43)

Paternal depressive symptoms were assessed *via* the Patient Health Questionnaire (PHQ-9; Kroenke et al., 2001), a self-administered, nine item measure of depression severity. Participants indicate the severity of depressive symptomology, such as “*little interest or pleasure in doing things*,” over the last 2 weeks through responses on a four-part Likert scale ranging from “*not at all*” to “*nearly every day*.” The PHQ-9, including its clinical cut-off points have been validated for sensitivity and specificity in the UK general population (Gilbody et al., 2006). Higher scores on the PHQ-9 are indicative of more depressive symptoms experienced in the last 2 weeks. The lowest possible score is zero, the highest is 27.

COVID-19 impact was assessed *via* the Coronavirus Impact Scale (CIS; National Institute of Environmental Health Sciences[NIEHS], 2020), an 11-item survey. The scale assesses the extent to which the COVID-19 pandemic affected an individual’s life across several domains including family income/employment, food access, mental healthcare access and stress/family discord. Responses range from “*mild*” to “*severe*” across a four-point Likert scale, with examples given pertinent to each domain, for example “*Mild. Occasional worries and/or minor stress-related symptoms (e.g., feel a little anxious, sad, and/or angry; mild/rare trouble sleeping)*.” No timeframe of reference was given for these questions, the inference being the individual reports based on their overall experience of the pandemic. A higher CIS suggested the COVID-19 pandemic had a greater impact on participants and their families. Scores ranged from zero to a possible 35 as the maximum score.

Child cognition was assessed using the Patient Report Outcomes Measurement Information System [PROMIS] (2019), Parent Proxy- Cognitive Function (PPCF; Lai et al., 2011). The PPCF is a parent report measure that probes child cognition in terms of mental acuity, memory, and verbal fluency. Participants were asked to indicate the frequency by which their child has difficulties with certain cognitive tasks, across 25 items, for example “*your child has trouble recalling the names of things*” over the last four weeks. Responses range from “*none of the time*” to “*all of the time*” across a five-point Likert scale. This measure was developed for use in children aged 5–17 years old, it has demonstrated excellent inter-rater reliability and ROC curves imply validity as well as clinical utility (Lai et al., 2011; Irwin et al., 2012). The higher the score on the PPCF, the better the perceived cognition of the child, thus a lower score indicates more deficits. The lowest possible score is 25, the highest 125.

Parenting behavior was assessed using the Parenting Style and Dimensions Questionnaire (PSDQ; Robinson et al., 2001), specifically the 15-item authoritative parenting subsection. The self-report items allowed an overall score of authoritative parenting to be generated by probing domains such as regulation support, autonomy granting and warmth. Responses indicated how often such specific parenting behaviors are demonstrated along the Likert scale “*never*” to “*always*.” The greater the score, the more prevalent the associated behaviors, the lowest possible score is 15 and the highest is 75. Each sub-set behavior dimension of the PSDQ has demonstrated excellent internal consistency (Robinson et al., 2001) and predictive validity (Olivari et al., 2013).

Child behavioral and emotional outcomes were assessed using the Strengths and Difficulties Questionnaire (SDQ; Goodman, 2001). The SDQ is a widely operationalized tool which measures child emotional and behavioral problems within clinical and non-clinical settings. The parent-report version consists of 25-items, responses are given along a three-part Likert scale with a top score of 50 and a bottom score of zero. Higher scores on the SDQ and its subsidiaries (internalizing; behavioral problems, and externalizing; emotional problems), indicate more difficulties. Cronbach’s α consistently judges the SDQ internal reliability as satisfactory (Goodman and Goodman, 2011).

### Qualitative data pertaining to paternal experiences of the pandemic

Following the survey items, an open-answer section was also included stating “*please provide any additional information relevant to this section*.” Where the questionnaire pertained to child experiences the question was amended slightly to, “*please provide any additional relevant information for this section, e.g., has there been a change in your child since the COVID-19 pandemic?*”

#### Data analysis

Data was analyzed using the IBM SPSS Statistics for Windows, Version 26.0 The available case method was utilized, meaning cases were only removed when they contained missing data from the variable of interest in any one analysis. As pairwise deletion was used, the *n* for each measure varies and is indicated in Table 2.

**TABLE 2:** Descriptive statistics and internal consistency for predictor and outcome variables.

Measure	*n*	*M*	*SD*	α
**Predictors**				
PHQ-9	85	5.55	4.96	0.86
CIS	79	20.97	4.91	0.78
**Outcomes**				
PPCF	80	108.80	14.29	0.95
PSDQ	82	3.95	0.52	0.88
SDQ total	77	1.92	1.10	0.73
SDQ internalizing	80	0.81	0.68	0.78
SDQ externalizing	80	1.15	0.65	0.70

PHQ-9, patient health questionnaire; CIS, coronavirus impact scale; PPCF, parent proxy cognitive function; PSDQ, parenting style and dimensions questionnaire; SDQ, strengths and difficulties questionnaire.

Two predictor variables were collected, COVID-19 impact and paternal depression. The outcome variables of focus were parenting behavior, child cognition, as well as emotional and behavioral difficulties. Correlations were run between all variables, including socioeconomic status (SES), as possible covariates. Additionally, multicollinearity was assessed but found to be within acceptable limits (Bowerman and O’Connell, 1990; Menard, 1995). Where a significant association was found, regression analyses were conducted to examine whether the correlated independent variables were significant predictors of cognitive deficit, parenting style and SDQ scores. Where a facet of SES was significantly correlated with an outcome variable, this was entered into the regression model as a covariate, hierarchically.

To investigate the paternal responses to the open-ended questions, content analysis was conducted independently by a single researcher (Chambers and Chiang, 2012). Codes (Table 8) were not predetermined; however initial labeling was guided by locating statements pertaining to reported changes in the lives of parents and children caused by the pandemic. Labeling was guided in this way because the open-ended questions often suggested this topic as an exemplar for answers. Codes were generated by the frequency in-which certain themes arose. These codes were then organized by commonalties into overarching categories which are reported.

**TABLE 3:** Predictor and outcome variable zero-order Pearson’s correlations.

Measure	1	2	3	4	5	6	7	8	9
**Predictor**									
PHQ-9	–								
CIS	0.58**	–							
**Outcome**									
PPCF	−0.54**	–0.48	–						
PSDQ	–0.10	−0.27**	−0.34**	−0.30**	–				
SDQ total	0.46**	0.56**	−0.69**	−0.64**	−0.32*	–			
SDQ internalizing	0.30**	0.39**	−0.53**	−0.50**	−0.28**	0.85**	–		
SDQ externalizing	0.42**	0.51**	−0.63**	−0.61**	−0.35**	0.87**	−0.49**	–	
**Covariates**									
SES education	–0.04	−0.23**	–0.01	0.06	0.05	–0.02	0.16	–0.13	–
SES income	−0.35**	−0.33**	0.07	–0.02	–0.16	–0.18	–0.08	–0.17	0.28**

*p < 0.05 and **p < 0.01 (1-tailed). PHQ-9, patient health questionnaire; CIS, coronavirus impact scale; PPCF, parent proxy cognitive function; PSDQ, parenting style and dimensions questionnaire; SDQ, strengths and difficulties questionnaire; SES, social economic status.

**TABLE 4:** Hierarchical multiple regression analysis predicting depressive symptom scores from COVID-19 impact, holding education and income constant.

	PHQ-9						
Predictor	*B*	*SE B*	β	*t*	*R* ^2^	Δ*R*^2^	Δ*F*
*Step one*					0.13	0.13	**5.52** **
Constant	18.38	2.05		8.97			
	[14.30, 22.46]						
SES education	0.27	0.44	0.07	0.60			
	[−0.61, 1.14]						
SES income	−0.65	0.20	–0.37	**−−3.30** **			
	[−1.05, −0.25]						
*Step two*					0.39	0.26	**31.05** **
Constant	3.51	3.18		1.10			
	[−2.82, 9.84]						
SES education	0.59	0.38	0.15	1.56			
	[−0.16, 1.33]						
SES income	−0.38	0.17	–0.22	**−−2.21** **			
	[−0.73, −0.04]						
CIS	0.55	0.10	0.55	**5.57** **			
	[0.35, 0.75]						

A 95% confidence intervals reported in parentheses. **p < 0.01. PHQ-9, patient health questionnaire; CIS, coronavirus impact scale; PPCF, parent proxy cognitive function; SES, social economic status. The bold values indicate their significance.

**TABLE 5:** Hierarchical multiple regression analysis predicting cognition scores from paternal depressive symptoms and COVID-19 impact, holding education and income constant.

	PPCF						
Predictor	*B*	*SE B*	β	*t*	*R* ^2^	Δ*R*^2^	Δ*F*
*Step one*					0.01	0.01	0.19
Constant	107.42	6.59		16.31			
	[94.28, 120.57]						
SES education	−0.37	1.41	−0.03	−0.26			
	[−3.18, 2.43]						
SES income	−0.38	0.63	0.08	0.61			
	[−0.87, 1.64]						
*Step two*					0.36	0.35	**18.24** **
Constant	141.77	10.12		13.92			
	[121.43, 162.113]						
SES education	−0.52	1.20	−0.05	−0.44			
	[−2.92, 1.87]						
SES income	−0.86	0.57	−0.17	1.51			
	[−1.20, 0.28]						
PHQ-9	−1.18	0.40	−0.41	**−2.25** **			
	[−1.98, −0.39]						
CIS	−0.84	0.37	−0.29	**−2.25** **			
	[−1.58, −0.09]						

A 95% confidence intervals reported in parentheses. **p < 0.01. PHQ-9, patient health questionnaire; CIS, coronavirus impact scale; PPCF, parent proxy cognitive function; SES, social economic status. The bold values indicate their significance.

**TABLE 6:** Hierarchical multiple regression analysis predicting parenting behavior from paternal COVID-19 impact scores, holding education, and income constant.

	PSDQ						
Predictor	*B*	*SE B*	β	*t*	*R* ^2^	Δ*R*^2^	Δ*F*
*Step one*					0.03	0.03	1.21
Constant	4.03	0.23		17.58			
	[3.58, 4.49]						
SES education	0.04	0.05	−0.18	0.82			
	[−0.06, 0.14]						
SES income	−0.03	0.02	−0.18	−1.50			
	[−0.08, 0.01]						
*Step two*					0.14	0.10	**8.47** **
Constant	5.02	0.40		12.49			
	[4.21, 5.82]						
SES education	0.02	0.05	0.05	0.40			
	[−0.08, 0.11]						
SES income	−0.05	0.02	−0.28	**−2.33** *			
	[−0.10, −0.01]						
CIS	−0.04	0.01	−0.35	−2.91**			
	[−0.06, −0.11]						

*p < 0.05 and **p < 0.01 (1-tailed). CIS, coronavirus impact scale; PSDQ, parenting style and dimensions questionnaire; SES, social economic status. The bold values indicate their significance.

**TABLE 7:** Hierarchical multiple regression analysis predicting SDQ scores from paternal depressive symptoms and COVID-19 impact, holding education and income constant.

	SDQ						
Predictor	*B*	*SE B*	β	*t*	*R* ^2^	Δ*R*^2^	Δ*F*
*Step one*					0.04	0.04	1.21
Constant	2.35	0.51		4.63			
	[1.34, 3.36]						
SES education	0.04	0.11	0.04	0.33			
	[−0.18, 0.25]						
SES income	−0.08	0.05	−0.20	−1.55			
	[−0.17, 0.02]						
*Step two*					0.35	0.32	**10.24** **
Constant	−0.95	0.80		−1.18			
	[−2.55, 0.65]						
SES education	0.09	0.09	0.10	0.93			
	[−0.10, 0.28]						
SES income	0.00	0.05	0.01	0.06			
	[−0.09, 0.09]						
PHQ-9	0.04	0.03	1.94	1.38			
	[−0.02, 0.11]						
CIS	0.11	0.03	0.48	**3.68** **			
	[0.05, 0.17]						

**p < 0.01 (1-tailed). PHQ-9, patient health questionnaire; CIS, coronavirus impact scale; SES, social economic status; SDQ, strengths and difficulties questionnaire. The bold values indicate their significance.

**TABLE 8:** Code category, ranking and frequency of factors impacted by the COVID-19 pandemic.

Ranking	Categories	Code count	Percentage
1	Concern for child	13	25.00
2	Work	11	21.15
3	No change	10	19.23
4	Mental health	8	15.38
5	Family contact	5	9.62
6	Positive experiences	5	9.62

### Ethical approval

Ethical approval was granted by the Research Integrity and Governance Office (RIGO), University of Surrey, UK.

## Results

Ninety participants took part, three of whom completed less than 70% of the survey; this data was excluded from analysis. As such, 87 fathers (*M*_*age*_ = 41.34, *SD* = 6.95) were available for analysis aged between 23 and 62 years. Paternal demographics are presented in Table 1. Of the fathers, 17 (20%) met the threshold for moderate depression or above (≥10). Participants’ children consisted of 52 boys (*M*_*age*_ = 8.04, *SD* = 1.76) and 35 girls (*M*_*age*_ = 8.43, *SD* = 1.60), aged 6–11 years old. Further descriptive data, including tests of internal consistency for each predictor and dependent variable are outlined in Table 2.

### Quantitative data

Zero-order correlations were conducted between each predictor variable, the dependent variables, and SES (Table 3). Paternal depressive symptoms and COVID-19 impact were positively correlated (*r* = 0.58, *p* ≤ 0.01). Further, paternal depressive symptoms (*r* = −0.54, *p* ≤ 0.01) and COVID-19 impact (*r* = 0.48, *p* ≤ 0.01) were correlated with more cognitive deficits in children. Paternal depressive symptoms were not correlated with parenting behavior but were positively associated with overall SDQ (*r* = 0.46, *p* ≤ 0.01) and both internalizing (*r* = 0.30, *p* ≤ 0.01) and externalizing (*r* = 0.42, *p* ≤ 0.01) subscales. Paternal income was negatively correlated with symptoms of depression (*r* = −0.35, *p* = ≤ 0.01). Paternal income (*r* = −0.33, *p* ≤ 0.01) and education (*r* = −0.23, *p* ≤ 0.01) was negatively associated with COVID-19 impact, as such these SES factors were entered into each regression as covariates.

### The impact of the pandemic on paternal depression

A hierarchical regression was undertaken to examine the relationship between COVID-19 impact scores and paternal depressive symptoms (Table 4). Paternal education and household income were held constant in the model and this step accounted for 13% of variation in depressive symptoms, [*F*_(2, 74)_ = 5.5, *p* ≤ 0.01]. The full model also reached significance, accounting for 39% variability [*F*_(3,73)_ = 0.15.53, *p* ≤ 0.01], with income (β = −0.22) and COVID-Impact (β = −0.55) each carrying a significant regression weight.

### The impact of paternal depression and the pandemic on child cognitive outcomes

A hierarchical regression model was utilized to examine the impact of paternal depression and the pandemic on child cognitive deficit scores (Table 5). Paternal qualification and household income accounted for less than 1% of variation in scores and were not significant [*F*_(2, 69)_ = 0.19, *p* = 0.83]. However, the full model reached significance, accounting for 35% additional variability in child cognition [*F*_(5, 65)_ = 7.21, *p* ≤ 0.01]. Paternal depressive symptoms (β = −0.41) and COVID-19 impact (β = −0.29) both independently carried a significant regression weight.

### The impact of the pandemic on paternal parenting behavior

Table 6 outlines the hierarchical regression exploring the variability accounted for in parenting style scores by COVID-19 impact scores. Paternal income and qualifications accounted for 3% of the variability in authoritative parenting scores, [*F*_(2, 71)_ = 1.21, *p* = 0.30] but did not reach significance. However, the full model was significant and accounted for 14% of scores [*F*_(3,70)_ = 4.07, *p* ≤ 0.01], with income (β = −0.28) and COVID-19 impact (β = −0.35) carrying a significant negative regression weight.

### The impact of paternal depression and the pandemic on child behavioral and emotional outcomes

A hierarchical regression was undertaken to determine if paternal depressive symptoms and pandemic-related impact significantly predict child emotional and behavioral scores as measured by the SDQ (Table 7). Paternal education and income were entered into step 1 and accounted for 4% of SDQ scores, [*F*_(2, 66)_ = 1.21, *p* = 0.30], not reaching significance. However, the full model was significant, accounting for 35% of variance [*F*_(4, 64)_ = 8.67, *p* ≤ 01]; COVID-impact scores carried a significant regression weight in a positive direction (β = 0.48), suggesting that as paternal difficulties related to the pandemic increased, perceived child emotional and behavioral difficulties rose concurrently.

### Qualitative data

Of the 87 participants, 33 (37.9%) responded to at least one of the open-ended questions asking fathers to expand on how life had changed due to the COVID-19 pandemic. Themes were drawn from these comments, and codes created on commonalities in concept and terminology, using content analysis. As outlined in Table 8, six categories were generated pertaining to changes and concerns resulting from the pandemic. Sub-themes and example quotes are also reported (Table 9) as well as brief descriptions and implications.

**TABLE 9:** Summary of sub-themes and example quotes.

Categories	Sub-themes	Example quotes
Concern for child	Anxiety; Schooling; Practical; Frustration; Physical health	“He is more anxious and less keen to go out;” “…Lack of stimulation was a struggle;” “My son has become very clingy to me fixated even;” “[My] child had COVID, feels tired a lot afterward”
Work	“Working from home;” “Increased workload;” “Travel”	“Increase [in] my workload;” “Not having the time or mental space to do my own work”
No change	Reports of no change; Ways of coping	“Not much change from before;” “No [change] because we don’t watch the news;” “None”
Mental health	Depression; Anxiety; Isolation	“Causing depression and anxiety;” “Bad for my mental health;”
Family contact	Unable to visit family	“Very limited contact with parents;” “Impossible to visit family;” “Not being able to see vulnerable relatives.”
Positive exp.	Family time; Flexibility	“It has meant more time in the house together so… an opportunity to have family time”

### Concern for child

Most fathers that responded noted a change in their child’s behavior, such as anxiety, “*increased anxiety, reluctant to sleep*,” confidence “*one child saw confidence drop a lot during COVID*,” and anger “*in lockdown he was prone to outbursts and mild violence.*” Interestingly, most comments that stated such behavior change also provided a solution-based approach or caveat to resolution, “*we have been able to help more intensely with schoolwork and learning*…*we’ve had to build* [confidence] *back up again*… *though this has subsided in recent weeks.*” This may provide some insight into one method fathers utilized to check their child’s wellbeing through the pandemic, monitoring for behavioral change, as well as their approach for managing said perceived change.

### Work

Fathers highlighted the challenges of balancing parenting whilst working from home, “*I am a full-time single father–so the greatest impact was home schooling.*” This also emphasized the disparate experiences of those with existing stressors during the pandemic, in this instance family structure resulted in one father feeling more vulnerable to difficulties in the context of homeworking. Other responders shared that the pandemic resulted in more work due to “*supporting* [employees] *through the pandemic*” and “*staff shortages*” because of illness.

### No change

Many fathers specifically stated that there were no perceived changes to their lives due to the pandemic. It is unclear from the provided responses what underpins this perception of “*no significant change*,” although it seemed to mostly pertain to their own experience. Only one father shared a possible coping mechanism linked to maintaining this position of no change, “*we don’t watch the news.*”

### Paternal mental health

This category consisted of comments explicitly made regarding mental health challenges, namely “*depression and anxiety*” as well as known precipitators to mental health difficulties, such as “*isolation.*” Those that responded also described the experience of lockdown, “*difficult and mentally hard on me*” with some indicating behavior change as a result “*I’ve been busy and distracted recently.*”

### Family contact

Fathers described the difficulties related with social isolation due the pandemic, “*feeling cut off from family members in Australia and New Zealand as both countries have implemented strict border closures for an extended period.*” They also shared worries and “*concern for family members*” because of the implications of catching COVD-19.

### Positive experiences

Benefits of lockdown mainly orientated around spending “*time with the kids which I enjoyed.*” It was mostly a reduction in time related to work which seemed to facilitate this change “*more family time as a consequence of working from home*… *no commute so more time with family.*” Although, one father also described “*survivors*’ *guilt as we* [were] *lucky enough to not have been affected as a family.*”

## Discussion

There is a paucity of research studying the effect of paternal depression on parenting behavior and child development. This is especially relevant given the global COVID-19 pandemic, which likely impacted on the lives of fathers and their mental health (Cheng et al., 2021). As such, this study aimed to explore the impact of the pandemic on fathers of children aged 6–11 years old, as well as paternal depressive symptoms on parenting behavior and child cognitive, emotional, and behavioral outcomes.

This study found that greater impact of COVID-19 scores and lower income were associated with higher levels of depressive symptoms in fathers. Pandemic impact and paternal depression were linked to higher levels of father-rated child cognitive deficits (in areas such as mental acuity, memory, and verbal fluency) as well as emotional and behavioral difficulties. Paternal COVID-19 impact scores, but not depressive symptoms, were associated with less authoritative parenting behaviors, characterized as warmth and autonomy granting. Qualitative responses from fathers highlighted worries about children, work, and mental health as most prevalent during the pandemic. However, several responders also noted no change during the pandemic, and some listed positive experiences during this time.

Fathers in this study appeared negatively impacted by the COVID-19 pandemic, self-reporting high levels of depression. Indeed, 20% of responders met threshold for moderate depression as rated by the PHQ-9. This is concerning when considering the literature regarding lack of paternal help seeking at times of distress (O’Brien et al., 2017; Schuppan et al., 2019). Evidence indicates that men are more likely to rely on harmful or perpetuating coping strategies, such as drugs and alcohol, avoidance, and suppression of emotion (O’Brien et al., 2017). This can result in paternal depression symptoms being misdiagnosed, wrongly attributed to environmental stressors, or missed entirely (Musser et al., 2013; O’Brien et al., 2017). Indeed, in a study of 406 British adults, 46.3% of participants correctly identified postnatal depression in fathers, compared to 90.1% when the targets were mothers (Swami et al., 2020). These findings suggest fathers are unlikely to express, and others unlikely to recognize paternal depression. This is serious in the context of the pandemic as undiagnosed, untreated depression can have severe consequences (Biddle et al., 2008; Quevedo et al., 2011), especially significant at a time when paternal contact with others would have been reduced significantly.

Qualitative studies indicate the main barrier to paternal support seeking is worries around perceptions of weakness and stigma (O’Brien et al., 2017; Schuppan et al., 2019). In this study, “no change” experienced during a global pandemic was a theme reported in the qualitative data. This may align with the theoretical position of gendered expression of difficulty, whereby fathers completing this study felt less able to share the distress caused by the pandemic. As hypothesized, both greater paternal pandemic impact and depressive symptoms were predictive of parent-proxy cognition scores in 6–11-year-old children. The underpinnings of this relationship are likely numerous and multi-faceted and need to be explored further in future research. For example, fathers impacted more by the pandemic may have felt less able to support their child through home-schooling, and as such perceive a detriment in their cognition.

One explanation evidenced in-part by this research, is the possibility that paternal depression and COVID-19 impact negatively influence paternal parenting behavior, which was in turn detrimental to child cognition. If this were the case, it calls into question the difference in how each predictor variable was related to parenting behavior scores. Pandemic impact, but not depressive symptoms predicted paternal authoritative parenting style. This may suggest that COVID-19-impact limited positive parenting behaviors in fathers such as warmth and autonomy-granting, which in-turn negatively impacted on child cognition scores. It is possible that another behavioral mechanism of action accounts for the relationship between paternal depression and perceived child cognitive deficits. For example, paternal depression has been found to result in more withdrawn, unresponsive behavior during father-child interaction (Dette-Hagenmeyer and Reichle, 2014; Sethna et al., 2015). This would many implications for cognitively stimulating behaviors, such as those related to paternal play.

Paternal depressive symptoms were related to parent-rated emotional and behavioral scores in children aged 6–11 years old. This is consistent with previous research which has found a link between depression in fathers and similar developmental outcomes across the lifespan (Gutierrez-Galve et al., 2015; Sweeney and MacBeth, 2016). This research expands on these findings by also implicating paternal negative experiences of the pandemic with child emotional and behavioral difficulties. One inference from such results could be that fathers perceived their child as struggling because of the pandemic. This is concordant with their reports in qualitative feedback, concern for child was the category coded for the most, with themes of child low confidence and anxiety.

### Clinical implications of this research

The results suggest that the pandemic is associated with higher levels of depressive symptoms in fathers. Those fathers who experienced depression during the pandemic, especially from low-income backgrounds, may represent an at-risk group who require additional support. This is an important consideration in terms of policy and aid distribution. Future studies may benefit from working with fathers to determine their depressive etiology in terms of terminology, coping and behavior. Indeed, their presentation is likely to differ greatly from mothers and psychoeducation on this delivered to healthcare networks as well as families would be beneficial (O’Brien et al., 2017).

Interventions for paternal depression are extremely limited. In a recent systematic review spanning 25 years of research, not a single randomized control trial had been produced which included an intervention solely targeting fathers (Goldstein et al., 2020). As the findings indicate additional COVID-19 related stress was associated with depression in fathers, stress management interventions may be a beneficial, low-cost intervention which could be explored in future research as a potential preventative. For example, mindfulness interventions have been shown to be effective in reducing parental stress, with a subsequent positive impact on youth outcomes (Burgdorf et al., 2019).

However, further support is needed to address paternal depression explicitly, especially given the findings of this study. In the UK, NICE (National Institute for Health and Care Excellence[NIHCE], 2009) have made recommendations for how to treat depression, yet there are no specific guidelines for fathers despite growing evidence that men may experience depression differently to women (Hambridge et al., 2021).

As well as fathers, considerations must be given to the findings of this study demonstrating an effect on children. For example, COVID-19 impact was negatively associated with paternal positive parenting behaviors. Relationship-based parenting interventions have been found effective in improving parenting-child relationships long-term (Mortensen and Mastergeorge, 2014; Madden et al., 2015; Duncan et al., 2017). Such interventions may be adaptable to consider post-pandemic father-child relationships.

The study suggests that children aged 6–11 years old who had a father experiencing depressive symptoms during the pandemic, may also be more vulnerable to negative developmental outcomes. School-based interventions which provide emotional support to children from a low-income background, living with a caregiver experiences symptoms of depression have been found effective (Johnson et al., 2013). Additional support should be offered to children who may be at risk due to familial hardship experienced during the pandemic.

### Limitations and future research

As a cross-sectional design was utilized, the findings from this study are limited in terms of direction. For example, many studies have suggested that a bidirectional impact exists between parents and child (Pearl et al., 2014; Thomason et al., 2014; Brinke et al., 2017). As such, it may be that challenging child behavior during the pandemic resulted in the pandemic feeling more impactful, as fathers were unable to engage with coping strategies outside the family home. To truly understand the bidirectional complexities of the family system, larger scale longitudinal research is needed.

Further, as this study utilized self-report measures and the relatively small sample size, the outcomes cannot be considered subjective and therefore limited in generalizability. However, as research is lacking from the perspective of fathers, it is useful to explore the paternal perceptions of the pandemic and their child’s development.

This study measured ethnic background, however lack of variability in the sample prevented further analysis. Despite this, the effect of culture and ethnicity on paternal mental health and parenting style should not be ignored. In an American population study of 5,088 family units, a comprehensive review of paternal-infant interaction by race/ethnicity found that African American and Latino fathers displayed higher levels of caregiving and play activities than White fathers, when familial status, income and mental health were held constant (Cabrera et al., 2011). Such findings signifies the need for the inclusion of culture and family structure (Ryan et al., 2015; Roman et al., 2016) in future models of child development.

## Conclusion

This study aimed to explore the relationship between the impact of the COVID-19 pandemic and paternal depression on parenting behavior and father-reported child developmental outcomes. Findings indicated that fathers’ higher impact of COVID-19 scores and lower paternal income predicted greater levels of paternal depressive symptoms. COVID-19 impact, and paternal depression were associated with higher levels of father-rated child cognitive deficits as well as emotional and behavioral difficulties. Paternal pandemic impact scores, but not depressive symptoms, predicted less positive authoritative parenting behaviors, characterized as lower levels of warmth and autonomy-granting. Qualitative responses from fathers suggested increased worries about their children, workload, and their own mental health during the pandemic. However, interestingly, several responders noted no change to their lives during the pandemic. The findings indicate a need for a greater understanding of depression in fathers, as well as interventions that support families through times of increased stress, such as during a pandemic.

## Data availability statement

The original contributions presented in this study are included in the article/supplementary material, further inquiries can be directed to the corresponding author.

## Ethics statement

The studies involving human participants were reviewed and approved by the Research Integrity and Governance Office (RIGO), University of Surrey. The patients/participants provided their written informed consent to participate in this study.

## Author contributions

JR, R-MS, and JI were involved in the conceptualization, design, and write-up of this study. JR collected the data, performed the statistical analysis, and wrote the first draft of the manuscript. All authors contributed to manuscript revision.

## References

[B1] Baker C. Kuhn L. (2018). Mediated pathways from maternal depression and early parenting to children’s executive function and externalising behaviour problems. *Infant Child Dev.* 27:e2052. 10.1002/icd.2052

[B2] Biddle L. Brock A. Brookes S. T. Gunnell D. (2008). Suicide rates in young men in england & wales in the 21st century: Time trend study. *Br. Med. J.* 336:536. 10.1136/bmj.39475.603935.25 18276666 PMC2265363

[B3] Blair C. (2016). Developmental science and executive function. *Curr. Direct. Psychol. Sci.* 25 3–7. 10.1177/0963721415622634 26985139 PMC4789148

[B4] Bowerman B. L. O’Connell R. T. (1990). *Linear Statistical Models: An Applied Approach*, 2nd Edn. Belmont, CA: Duxbury.

[B5] Brinke L. W. Dekovic M. Stoltz S. E. Cilessen A. H. (2017). Bidirectional effects between parenting & aggressive child behaviours in the context of preventive intervention. *J. Abnorm. Child Psychol.* 45 921–934. 10.1007/s10802-016-0211-3 27787671 PMC5487807

[B6] Burgdorf V. Szabo M. Abbott M. J. (2019). The effect of mindfulness intervention for parents on parenting stress and youth psychological outcomes: A systematic review and meta-analysis. *Front. Psychol*. 10:1336. 10.3389/fpsyg.2019.01336 31244732 PMC6562566

[B7] Cabrera N. J. Fitzgerald H. E. Bradley R. H. Roggman L. (2014). The ecology of father–child relationships: An expanded model. *J. Fam. Theory Rev.* 6 336–354.

[B8] Cabrera N. J. Hofferth S. L. Chae S. (2011). Patterns and predictors of father-infant engagement across race/ethnic Groups. *Early Child. Res. Q.* 26 365–375. 10.1016/j.ecresq.2011.01.001 22110258 PMC3220616

[B9] Cabrera N. J. Shannon J. Tamis-LeMonda C. (2007). Fathers’ Influence on their children’s cognitive and emotional development: From toddlers to pre-K. *Appl. Dev. Sci.* 11 208–213. 10.1080/10888690701762100

[B10] Cabrera N. J. Volling B. L. Barr R. (2018). Fathers are Parents, Too! Widening the Lens on Parenting for Children’s Development. *Child. Dev. Perspect.* 12 152–156. 10.1111/cdep.12275

[B11] Chambers T. Chiang C. H. (2012). Understanding undergraduate students’ experience: A content analysis using NSSE open-ended comments as an example. *Qual. Quant.* 46 1113–1123. 10.1007/s11135-011-9549-3

[B12] Charrois J. Cote S. M. Paqui S. Seguin J. R. Japel C. Vitaro F. (2020). Maternal depression in early childhood and child emotional and behavioural outcomes at school age: Examining the roles of preschool childcare quality and current maternal depression symptomatology. *Eur. Child. Adolesc. Psychiatry* 29 637–648. 10.1007/s00787-019-01385-7 31410578

[B13] Cheng Z. Mendolia S. Paloyo A. R. Savage D. A. Tani M. (2021). Working parents, financial insecurity, and childcare: Mental health in the time of COVID-19 in the UK. *Rev. Econ. Household* 19 123–144. 10.1007/s11150-020-09538-3 33456425 PMC7802611

[B14] Cito G. Micelli E. Cocci A. Polloni G. Coccia M. E. Carini M. (2020). Paternal behaviours in the era of COVID-19. *World J. Men’s Health* 38 251–253. 10.5534/wjmh.200071 32378371 PMC7308232

[B15] Davison K. K. Charles J. N. Khandpur N. Nelson T. J. (2017). Fathers’ perceived reasons for their underrepresentation in child health research and strategies to increase their involvement. *Matern. Child. Health J.* 21 267–274. 10.1007/s10995-016-2157-z 27473093 PMC5500207

[B16] Dette-Hagenmeyer D. R. Reichle B. (2014). Parenting’s depressive symptoms and children’s adjustment over time are mediated by parenting, but differentially for father’s and mothers. *Eur. J. Dev. Psychol.* 11 196–210. 10.1080/17405629.2013.848789

[B17] Duncan K. M. MacGillivray S. Renfrew M. J. (2017). Costs & savings of parenting interventions: Results of a systematic review. *Child* 43 797–811. 10.1111/cch.12473 28557011

[B18] Feldman D. H. (2005). Paiget’s stages: The unfinished symphony of cognitive development. *N. Ideas Psychol.* 22 175–231. 10.1016/j.newideapsych.2004.11.005

[B19] Fletcher R. J. St George J. Freeman E. (2011). Rough and tumble play quality: Theoretical foundations for a new measure of father-child interaction. *Early Child. Dev. & Care* 183 746–759. 10.1080/03004430.2012.723439

[B21] Fredrikson E. Von Soest T. Smith L. Moe V. (2019). Parenting stress plays a mediating role in the prediction of early child development from both parents’ perinatal depressive symptoms. *J. Abnorm. Child Psychol.* 47 149–164. 10.1007/s10802-018-0428-4 29623542

[B22] Gentile S. Fusco M. L. (2017). Untreated perinatal paternal depression: Effects on offspring. *Psychiatry Res.* 252 325–332. 10.1016/j.psychres.2017.02.064 28314228

[B23] Gilbody S. Richards D. Barkham M. (2006). Diagnosing depression in primary care using self-completed instruments: UK validation of PHQ-9 and CORE-OM. *Br. J. General Pract.* 57 650–652. 17688760 PMC2099671

[B24] Glasser S. Lerner-Geva L. (2018). Focus on fathers: Paternal depression in the perinatal period. *Perspect. Public Health* 139 195–198. 10.1177/1757913918790597 30044191

[B25] Goldstein Z. Rosen B. Howlett A. Anderson M. Herman D. (2020). Interventions for paternal perinatal depression: A systematic review. *J. Affect. Disord.* 265 505–510. 10.1016/j.jad.2019.12.029 32090778

[B26] Goodman A. Goodman R. (2011). Population mean scores predict child mental disorder rates: Validating SDQ prevalence estimators in Britain. *J. Child Psychol. Psychiatry* 52 100–108. 10.1111/j.1469-7610.2010.02278.x 20722879

[B27] Goodman R. (2001). Psychometric properties of the strengths and difficulties questionnaire. *J. Am. Acad. Child. Adolesc. Psychiatry* 40 1337–1345. 10.1111/j.1469-7610.2010.02278.x 11699809

[B28] Gutierrez-Galve L. Stein A. Hanington L. Heron J. Ramchandani J. (2015). Paternal depression in the postnatal period and child development: Mediators and moderators. *Pediatrics* 135 339–347. 10.1542/peds.2014-2411 25560437

[B29] Hambridge S. Cowell A. Arden-Close E. Mayers A. (2021). What kind of man get depressed after having a baby?” fathers’ experiences of mental health during the perinatal period. *BMC Pregnancy Childbirth* 21:463. 10.1186/s12884-021-03947-7 34187395 PMC8244226

[B30] Irwin D. E. Gross H. E. Stucky B. D. Thissen D. DeWitt E. M. Lai J. S. (2012). Development of six promise pediatrics proxy-report item banks. *Health Qual. Life Outcomes* 10:22. 10.1186/1477-7525-10-22 22357192 PMC3312870

[B31] Jeynes W. H. (2016). Meta-analysis on the roles of fathers in parenting: Are they unique. *Marriage Fam. Rev.* 52 665–688. 10.1080/01494929.2016.1157121

[B32] John A. Halliburton A. Humphrey J. (2013). Child-mother & child-father play interaction patterns with pre-schoolers. *Early Child. Dev. Care* 183 483–497. 10.1080/03004430.2012.711595

[B33] Johnson S. R. Seidenfeld A. M. Izard C. E. Kobak R. (2013). Can classroom emotional support enhance prosocial development among children with depressed caregivers? *Early Child Res. Quart.* 28 282–290. 10.1016/j.ecresq.2012.07.003

[B34] Kroenke K. Spitzer R. L. Williams J. B. W. (2001). The PHQ-9 validity of a brief depression severity measure. *J. General Int. Med.* 16 606–613. 10.1046/j.1525-1497.2001.016009606.x 11556941 PMC1495268

[B35] Lai J. S. Butt Z. Zelko F. Cella D. Krull K. R. Kieran M. W. (2011). Developmental of a parent-report cognitive function item bank using response theory and exploration of it clinical utility in computerized adaptive testing. *J. Paediatr. Psychol.* 36 766–779. 10.1093/jpepsy/jsr005 21378106 PMC3146757

[B36] Liu Y. Kaaya S. Chai J. McCoy D. C. Surkan P. J. Black M. M. (2017). Maternal depressive symptoms and early childhood cognitive development: A meta-analysis. *Psychol. Med.* 47 680–689. 10.1017/S003329171600283X 27834159

[B37] Madden V. Domoney J. Auymayer K. Sethna V. Iles J. Hubbard I. (2015). International transmission of parenting: Findings from a uk longitudinal study. *Eur. J. Public Health* 25 1030–1035. 10.1093/eurpub/ckv093 26037954 PMC4668327

[B38] Martiny S. E. Thorsteinsen K. Parks-Stamm E. J. Olsen M. Kvalo M. (2021). Children’s wellbeing during the covid-19 pandemic: Relationships with attitudes, family structure, and mothers’ wellbeing. *Eur. J. Dev. Psychol.* 19 1–21. 10.1080/17405629.2021.1948398

[B39] McMunn A. Martin P. Kelly Y. Sacker A. (2017). Fathers’ involvement: Correlates and consequences for child socioemotional behaviour. *J. Fam. Issues* 38 1109–1131. 10.1177/0192513X15622415 28503014 PMC5418933

[B40] Menard S. (1995). *Applied Logistic Regression Analysis. Sage University Paper Series on Quantitative Applications in Social Sciences, 07-106.* Thousand Oaks, CA: Sage.

[B41] Mills-Koonce W. R. Willoughby M. T. Zvara B. Barnett M. Gustagsson H. Cox M. J. (2015). Mothers’ and fathers’ sensitivity and children’s cognitive development in low income, rural families. *J. Appl. Dev. Psychol.* 38 1–10. 10.1016/j.appdev.2015.01.001 25954057 PMC4418032

[B42] Mortensen J. A. Mastergeorge A. M. (2014). A meta-analytic review of relationship-based interventions for low-income families with infants & toddlers: Facilitating supportive parenting-child interactions. *Infant Ment. Health J.* 35 336–353. 10.1002/imhj.21451 25798486

[B43] Musser A. K. Ahmend A. H. Foli K. J. Coddington J. A. (2013). Paternal postpartum depression: What health care providers should know. *J. Pediatr. Health Care* 27 479–485. 10.1016/j.pedhc.2012.10.001 23182851

[B44] National Institute for Health and Care Excellence[NIHCE], (2009). *Depression in Adults: Recognition & Management.* Available online at: https://www.nice.org.uk/guidance/cg90 (accessed July, 2020). 31990491

[B45] National Institute of Environmental Health Sciences[NIEHS] (2020). *COVID-19 OBSSR Research Tools.* Available online at: https://www.nlm.nih.gov/dr2/COVID-19_BSSR_Research_Tools.pdf (accessed July, 2020).

[B46] O’Brien A. P. McNeil K. A. Fletcher R. Conrad A. Wilson A. J. Jones D. (2017). New fathers’ perinatal depression & anxiety – treatment options: An integrative review. *Am. J. Men’s Health* 11 863–876. 10.1177/1557988316669047 27694550 PMC5675308

[B47] Olivari M. G. Tagliabue S. Confalonieri E. (2013). Parenting styles and dimensions questionnaire: A review of reliability and validity. *Marriage Fam. Rev.* 49 465–490. 10.1080/01494929.2013.770812

[B48] Papaleontiou-Louca E. Omari O. A. (2020). The (neglected) role of the father in children’s mental health. *New Ideas Psychol*. 59:100782. 10.1016/j.newideapsych.2020.100782

[B49] Patrick S. Henkhaus L. E. Zickafoose J. Lovell K. Halvorson A. Loch S. (2020). Well-being of parents and children during the covid-19 pandemic: A national survey. *Paediatrics* 146:e2020016824. 10.1542/peds.2020-016824 32709738

[B50] Pearl A. French B. F. Dumas J. E. Moreland A. Prinz R. (2014). Bidirectional effects of parenting quality & child externalising behaviour in predominantly single parent, under-resourced African American families. *J. Child Fam. Stud.* 23 177–188. 10.1007/s10826-012-9692-z

[B51] Patient Report Outcomes Measurement Information System [PROMIS] (2019). *Cognitive Function: A Brief Guide to the PROMIS Cognitive Function Instruments.* Available online at: https://www.healthmeasures.net/search-view-measures?task=Search.search (accessed July, 2020).

[B52] Quevedo L. Azevedo da Silva R. Coelho F. Pinheiro K. A. T. Horta B. L. Kapczinski P. (2011). Risk of suicide & mixed episode in men in the postpartum period. *J. Affect. Disord.* 132 243–246. 10.1016/j.jad.2011.01.004 21277023

[B53] Robinson C. C. Mandleco B. Olsen S. F. Hart C. H. (2001). “The Parenting Styles and Dimensions Questionnaire (PSDQ),” in *Handbook of Family Measurement Techniques*, Vol. 3 eds Perlmutter B. F. Touliatos J. Holden G. W. (Thousand Oaks, CA: Sage), 319–321.

[B54] Robinson E. L. St George J. Freeman E. E. (2021). A systematic review of father-child play interactions and the impacts on child development. *Children* 8:389. 10.3390/children8050389 34068176 PMC8153002

[B55] Roggman L. A. Boyce L. K. Cook G. A. Christiansen K. Jones D. (2004). Playing with daddy: Social toy play, early head start, and developmental outcomes. *Fathering* 2 83–108. 10.3149/fth.0201.83

[B56] Rolle L. Gullotta G. Trombetta T. Curti L. Gerino E. Brustia P. (2019). Father involvement and cognitive development in early and middle childhood: A systematic review. *Front. Psychol.* 10:20405. 10.3389/fpsyg.2019.02405 31708843 PMC6823210

[B57] Roman N. V. Makwakwa T. Lacante M. (2016). Perceptions of parenting styles in south africa: The effects of gender and ethnicity. *Cogent Psychol.* 3:1153231. 10.1080/23311908.2016.1153231

[B58] Ryan R. M. Claessens A. Markowitz A. J. (2015). Associations between family structure change and child behaviour problems: The moderating effect of family income. *Child. Dev.* 86 112–127. 10.1111/cdev.12283 25209138

[B59] Sarkadi A. Kristiansson R. Oberklaid F. Bremberg S. (2008). Fathers’ involvement and children’s developmental outcomes: A systematic review of longitudinal studies. *Acta Paediatrica* 97 153–158. 10.1111/j.1651-2227.2007.00572.x 18052995

[B60] Schmidt A. Kramer A. C. Brose A. Schmiedek F. Neubauer A. B. (2021). Distance learning, parent-child interactions and affective well-being of parents & child during the COVID-19 pandemic: A daily diary study. *Dev. Psychol.* 57 1719–1734. 10.1037/dev0001232 34807692

[B61] Schuppan K. M. Roberts R. Powrie R. (2019). Paternal perinatal mental health: At risk father’s perceptions of help-seeking & screening. *J. Men’s Stud.* 27 307–328. 10.1177/1060826519829908

[B62] Sethna V. Murray L. Nesi E. Psychogiou L. Ramchandani P. G. (2015). Paternal depression in the postnatal period and early father-infant interactions. *Parent. Sci. Pract.* 15 1–8. 10.1080/15295192.2015.992732 25745364 PMC4337747

[B63] Swami V. Barron D. Smith L. Furnham A. (2020). Mental health literacy of maternal and paternal postnatal (postpartum) depression in British adults. *J. Ment. Health* 29 217–224. 10.1080/09638237.2019.1608932 31070064

[B64] Sweeney S. MacBeth A. (2016). The effects of paternal depression on child & adolescent outcomes: A systematic review. *J. Affect. Disord.* 205 44–59. 10.1016/j.jad.2016.05.073 27414953

[B65] Thomason E. Volling B. L. Flynn H. A. McDonough S. C. Marcus S. M. Lopez J. F. (2014). Parenting stress and depressive symptoms in postpartum mothers: Bidirectional or unidirectional effects? *Infant Behav. Dev.* 37 406–415. 10.1016/j.infbeh.2014.05.009 24956500 PMC4353594

[B66] Villodas M. T. Bagner D. M. Thompson R. (2015). A step beyond maternal depression and child behaviour problems: The role of mother-child aggression. *J. Clin. Child Adolesc. Psychol.* 47 634–641. 10.1080/15374416.2015.1094740 26672640

[B67] Waber D. De Moor C. Forbers P. Almli C. Botternon K. Leonard G. (2007). The NIH MRI study of normal brain development: Performance of a population based sample of health children aged 6 to 18 years on a neuropsychological battery. *J. Int. Neuropsychol. Soc.* 13 729–746. 10.1017/S1355617707070841 17511896

[B68] Waerden J. Galera C. Larroque B. Saurel-Cubizolles M. J. Sutter-Dallay A. L. Melchior M. (2015). Maternal depression trajectories and children’s behaviour at age 5 years. *J. Pediatr.* 166 1440–1448. 10.1016/j.jpeds.2015.03.002 25866387

[B69] Yogman M. Garfield C. F. (2016). Fathers’ roles in the care and development of their children: The role of paediatricians. *Am. Acad. Pediatr.* 138:e20161128. 10.1542/peds.2016-1128 27296867

